# Supplementary material for: Self-beneficial belief updating as a coping mechanism for stress-induced negative affect
Source: Sci Rep. 2021 Aug 24;11:17096. doi: 10.1038/s41598-021-96264-0 (PMC8384941; doi:10.1038/s41598-021-96264-0)
Supplement: Supplementary file 1 — Supplementary Information. [file 41598_2021_96264_MOESM1_ESM.docx]

**Supplementary Information**

**Title:** Self-beneficial belief updating as a coping mechanism for stress-induced negative affect

**Authors:**

Nora Czekalla^1^, Janine Stierand^1^, David S. Stolz^1^, Annalina V. Mayer^1^, Johanna F. Voges^1^, Lena Rademacher^1^, Frieder M. Paulus^1^, Sören Krach^1^, Laura Müller-Pinzler^1^

**Affiliations:**

^1^Social Neuroscience Lab at the Translational Psychiatry Unit (TPU), Department of Psychiatry and Psychotherapy, University of Lübeck, Ratzeburger Allee 160, D-23538 Lübeck, Germany

***Email addresses of corresponding authors:**

Nora Czekalla n.czekalla@uni-luebeck.de

Laura Müller-Pinzler laura.muellerpinzler@uni-luebeck.de

***Postal address of corresponding Authors:**

Nora Czekalla &Laura Müller-Pinzler

Department of Psychiatry and Psychotherapy,

Center of Brain, Behavior, and Metabolism (CBBM)

University of Lübeck, Ratzeburger Allee 160, D-23538 Lübeck, Germany

Phone: +49 451 3101 7510

**Supplementary Results**

| *Table S1.*  *Cortisol response -* *Scheirer-Ray-Hare Test* | | | | | | | | |
| --- | --- | --- | --- | --- | --- | --- | --- | --- |
|  | *Sum of Squares* | | *df* | | *H* | | *p* | |
| Stress group | 12642 | 2 | | 18.939 | | < .001 | |  |
| Time of the day | 6830 | 2 | | 10.232 | | .006 | |  |
| Stress group x Time of the day | 3640 | 4 | | 5.902 | | .207 | |  |
| Residuals | 35329 | 80 | |  | | |  | |
| *Note.* Group comparison of the stress-induced cortisol response (post-stress T2_CORT_ - baseline T1); *df* = degrees of freedom; *H* = test statistic; factor Stress group: social-evaluative stress (*n* = 29) vs. physical stress (*n* = 30) vs. no stress (*n* = 30), factor time of the day: morning vs. noon vs. afternoon. | | | | | | | | |

**Cortisol response**

**Negative affect ratings**

**
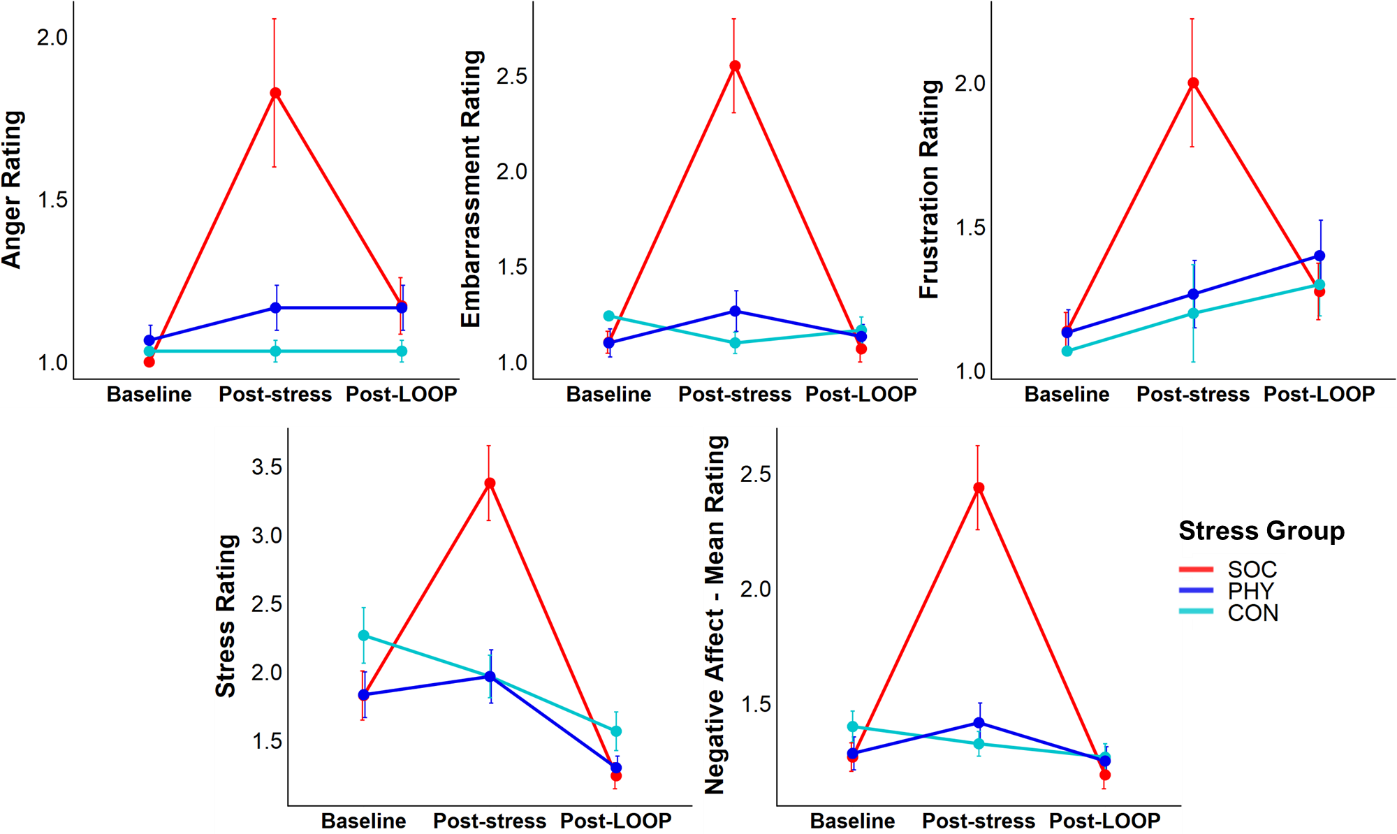
**

**Fig. S1**. Means and standard errors for the negative affect ratings separately for the three stress groups (SOC = social-evaluative stress [*n* = 29], PHY = physical stress [*n* = 30], CON =control [*n* = 30, embarrassment and frustration: *n* = 29 due to missing values]); LOOP = Learning of own performance task.

**Forming self-related beliefs over time - Model free behavior analysis**

| *Table S2. Performance Expectation Ratings - Linear model* | | | | | | | | | | | | | | |
| --- | --- | --- | --- | --- | --- | --- | --- | --- | --- | --- | --- | --- | --- | --- |
|  | |  | *B [95 % CI]* | | | *SE* | | *df* | | *t* | | *p* | | |
| Intercept | | | | 42.33  [40.48; 44.19] |  | 0.95 |  | 5156 |  | 44.70 |  | < .001 |  |  |
| Ability condition | | | | 9.59  [7.36; 11.83] |  | 1.13 |  | 86 |  | 8.52 |  | <.001 |  |  |
| Ability condition ✻  SOC vs. [PHY, CON] | | | | -0.35  [-1.94; 1.24] |  | 0.80 |  | 86 |  | -0.43 |  | .665 |  |  |
| Ability condition ✻ PHY vs. CON | | | | 0.79  [-1.93; 3.52] |  | 1.37 |  | 86 |  | 0.58 |  | .564 |  |  |
| Trial | | | | -0.41  [-0.44; -0.37] |  | 0.02 |  | 5156 |  | -23.75 |  | < .001 |  |  |
| Trial ✻ SOC vs. [PHY, CON] | | | | -0.01 [-0.04; 0.01] |  | 0.01 |  | 5156 |  | -0.96 |  | .335 |  |  |
| Trial ✻ PHY vs. CON | | | | 0.02  [-0.02; 0.06] |  | 0.02 |  | 5156 |  | 1.13 |  | .261 |  |  |
| Trial ✻ Ability condition | | | | 0.80  [0.75; 0.84] |  | 0.02 |  | 5156 |  | 32.73 |  | < .001 |  |  |
| Trial ✻ Ability condition ✻ SOC vs. [PHY, CON] | | | | 0.07  [0.04; 0.10] |  | 0.02 |  | 5156 |  | 4.01 |  | < .001 |  |  |
| Trial ✻ Ability condition ✻ PHY vs. CON | | | | -0.06  [-0.12; -0.01] |  | 0.03 |  | 5156 |  | -2.15 |  | .031 |  |  |
| SOC vs. [PHY, CON] | | | | 0.96  [-0.38; 2.30] |  | 0.67 |  | 86 |  | 1.43 |  | .157 |  |  |
| PHY vs. CON | | | | -0.48  [-2.77; 1.81] |  | 1.15 |  | 86 |  | -0.42 |  | .677 |  |  |
|  | Note. Linear mixed-effects model fit by maximum likelihood; dependent variable: performance expectation ratings; continuous variable: Trial, factor variables: Ability condition (high vs. low) and Stress group (SOC = social-evaluative stress [*n* = 29], PHY= physical stress [*n* = 30], CON = control [*n* = 30]) split in the contrasts SOC vs. [PHY,CON] and PHY vs. CON; *B* = unstandardized beta coefficient; *CI* = 95 % confidence interval; *SE* = standard error of B; *df* = degrees of freedom. | | | | | | | | | | | | |  |

**Model Selection**

| \| *Table S3. Model comparison* \| \| \| \| \| \| \| \| \| \| \| \| --- \| --- \| --- \| --- \| --- \| --- \| --- \| --- \| --- \| --- \| --- \| \| *Model* \|  \| *PSIS-LOO* \| \| *LOO-SE* \| \| *LOO-Diff*  *(SE-Diff)* \| *% of* $\hat{k}$ *> 0.7* \| \| *No. Est. Parameters* \| \| \|  \|  \|  \| \|  \| \|  \|  \| \|  \| \| \| **Whole Sample** \| \|  \| \|  \| \|  \|  \| \|  \| \| \|  \| Unity Model \| -2028.5 \| 257.0 \| \| 267.1 (52.0) \| \| 0.09 \| 3 \| \| \|  \| Ability Model \| -1884.4 \| 247.4 \| \| 123.0 (95.9) \| \| 0.53 \| 4 \| \| \|  \| Valence Model \| -1761.4 \| 280.4 \| \|  \| \| 0.17 \| 4 \| \| \|  \| Mean Model \| -2531.9 \| 219.2 \| \| 770.5 (93.5) \| \| 0.00 \| 2 \| \| \| **Social-evaluative Stress** \| \|  \|  \| \|  \| \|  \|  \| \| \|  \| Unity Model \| -625.3 \| 83.1 \| \| 60.7 (21.4) \| \| 0.17 \| 3 \| \| \|  \| Ability Model \| -605.1 \| 91.8 \| \| 40.5 (16.4) \| \| 0.80 \| 4 \| \| \|  \| Valence Model \| -564.6 \| 91.7 \| \|  \| \| 0.29 \| 4 \| \| \|  \| Mean Model \| -877.4 \| 94.0 \| \| 312.7 (40.3) \| \| 0.00 \| 2 \| \| \| **Physical Stress** \| \|  \|  \| \|  \| \|  \|  \| \| \|  \| Unity Model \| -840.1 \| 225.7 \| \| 107.6 (43.3) \| \| 0.00 \| 3 \| \| \|  \| Ability Model \| -782.9 \| 208.8 \| \| 50.5 (62.1) \| \| 0.39 \| 4 \| \| \|  \| Valence Model \| -732.5 \| 247.5 \| \|  \| \| 0.11 \| 4 \| \| \|  \| Mean Model \| -905.5 \| 181.1 \| \| 173.1 (75.1) \| \| 0.00 \| 2 \| \| \| **Control** \| \|  \|  \| \|  \| \|  \|  \| \| \|  \| Unity Model \| -563.1 \| 92.2 \| \| 98.7 (19.9) \| \| 0.11 \| 3 \| \| \|  \| Ability Model \| -496.4 \| 96.8 \| \| 32.1 (17.3) \| \| 0.40 \| 4 \| \| \|  \| Valence Model \| -464.3 \| 98.3 \| \|  \| \| 0.11 \| 4 \| \| \|  \| Mean Model \| -749.0 \| 84.6 \| \| 284.7 (35.3) \| \| 0.00 \| 2 \| \| \|  \|  \|  \| \|  \| \|  \|  \| \|  \| \| \| *Note.* LOO = sum PSIS-LOO, approximate leave-one-out cross-validation (LOO) using Pareto-smoothed importance sampling (PSIS); LOO-SE = Standard error of PSIS-LOO; LOO-Diff (SE-Diff) = Difference in expected predictive accuracy (PSIS-LOO) for all models from the model with the highest PSIS-LOO (Valence Model) and standard errors of differences; percentage of $\hat{k}$ - estimated shape parameters of the generalized Pareto distribution - exceeding 0.7 (all according to Vehtari et al.^1^); No. Est. Parameters = number of estimated parameters in the model; social-evaluative stress (*n* = 29), physical stress (*n* = 30), control (*n* = 29). \| \| \| \| \| \| \| \| \| \| \| |
| --- | --- | --- | --- | --- | --- | --- | --- | --- | --- | --- | --- | --- | --- | --- | --- | --- | --- | --- | --- | --- | --- | --- | --- | --- | --- | --- | --- | --- | --- | --- | --- | --- | --- | --- | --- | --- | --- | --- | --- | --- | --- | --- | --- | --- | --- | --- | --- | --- | --- | --- | --- | --- | --- | --- | --- | --- | --- | --- | --- | --- | --- | --- | --- | --- | --- | --- | --- | --- | --- | --- | --- | --- | --- | --- | --- | --- | --- | --- | --- | --- | --- | --- | --- | --- | --- | --- | --- | --- | --- | --- | --- | --- | --- | --- | --- | --- | --- | --- | --- | --- | --- | --- | --- | --- | --- | --- | --- | --- | --- | --- | --- | --- | --- | --- | --- | --- | --- | --- | --- | --- | --- | --- | --- | --- | --- | --- | --- | --- | --- | --- | --- | --- | --- | --- | --- | --- | --- | --- | --- | --- | --- | --- | --- | --- | --- | --- | --- | --- | --- | --- | --- | --- | --- | --- | --- | --- | --- | --- | --- | --- | --- | --- | --- | --- | --- | --- | --- | --- | --- | --- | --- | --- | --- | --- | --- | --- | --- | --- | --- | --- | --- | --- | --- | --- | --- | --- | --- | --- | --- | --- | --- | --- | --- | --- | --- | --- | --- | --- | --- | --- | --- | --- | --- | --- | --- | --- | --- | --- | --- | --- | --- | --- | --- | --- | --- | --- | --- | --- | --- | --- | --- | --- | --- | --- | --- | --- | --- | --- | --- | --- | --- | --- | --- | --- | --- | --- | --- | --- | --- | --- | --- | --- | --- | --- | --- | --- | --- | --- | --- | --- | --- | --- | --- | --- | --- | --- |

**Posterior predictive checks: Behavioral analyses on the predicted data.**

| *Table S4. Predicted Performance Expectations - Linear model* | | | | | | | | | | | | | | |
| --- | --- | --- | --- | --- | --- | --- | --- | --- | --- | --- | --- | --- | --- | --- |
|  | |  | *B [95 % CI]* | | | *SE* | | *df* | | *t* | | *p* | | |
| Intercept | | | | 42.56  [40.80; 44.33] |  | 0.90 |  | 5098 |  | 47.13 |  | < .001 |  |  |
| Ability condition | | | | 9.05  [7.01; 11.08] |  | 1.03 |  | 85 |  | 8.82 |  | <.001 |  |  |
| Ability condition ✻  SOC vs. [PHY, CON] | | | | -0.03  [-2.92; 2.86] |  | 1.45 |  | 85 |  | -0.02 |  | .983 |  |  |
| Ability condition ✻ PHY vs. CON | | | | 1.30  [-1.56; 4.17] |  | 1.44 |  | 85 |  | 0.90 |  | .369 |  |  |
| Trial | | | | -0.42  [-0.44; -0.41] |  | 0.01 |  | 5098 |  | -49.37 |  | < .001 |  |  |
| Trial ✻ SOC vs. [PHY, CON] | | | | 0.00 [-0.02; 0.03] |  | 0.01 |  | 5098 |  | 0.40 |  | .690 |  |  |
| Trial ✻ PHY vs. CON | | | | 0.03  [0.01; 0.06] |  | 0.01 |  | 5098 |  | 2.83 |  | .005 |  |  |
| Trial ✻ Ability condition | | | | 0.84  [0.82; 0.87] |  | 0.01 |  | 5098 |  | 69.66 |  | < .001 |  |  |
| Trial ✻ Ability condition ✻ SOC vs. [PHY, CON] | | | | 0.07  [0.03; 0.10] |  | 0.02 |  | 5098 |  | 3.93 |  | < .001 |  |  |
| Trial ✻ Ability condition ✻ PHY vs. CON | | | | -0.10  [-0.13; -0.07] |  | 0.02 |  | 5098 |  | -5.85 |  | < .001 |  |  |
|  | Note. Linear mixed-effects model fit by maximum likelihood; dependent variable: performance expectations predicted by winning model; continuous variable: Trial, factor variables: Ability condition (high vs. low) and Stress group (SOC = social-evaluative stress [*n* = 29], PHY= physical stress [*n* = 30], CON = control [*n* = 29]) split in the contrasts SOC vs. [PHY,CON] and PHY vs. CON; *B* = unstandardized beta coefficient; *CI* = 95 % confidence interval; *SE* = standard error of B; *df* = degrees of freedom. | | | | | | | | | | | | |  |

**Learning parameters.**

| *Table S5. Learning rates - Linear model* | | | | | | | | | | | | | | | | | | | |  | |
| --- | --- | --- | --- | --- | --- | --- | --- | --- | --- | --- | --- | --- | --- | --- | --- | --- | --- | --- | --- | --- | --- |
|  | |  | | *B [95 % CI]* | | *SE* | | | *b* | | | *df* | | | *t* | | | *p* | |  | |
| Intercept | | | 0.091  [0.079; 0.105] | |  | | 0.007 |  | |  |  | | 85 |  | | 13.412 |  | | < 0.001 |  |  |
| PE-Valence | | | -0.013  [-0.020; -0.006] | |  | | 0.004 |  | | -0.178 |  | | 85 |  | | -3.596 |  | | < .001 |  |  |
| SOC vs. [PHY, CON] | | | 0.015  [-0.004; 0.034] | |  | | 0.010 |  | | 0.138 |  | | 85 |  | | 1.500 |  | | .137 |  |  |
| PHY vs. CON | | | -0.007  [-0.023; 0.010] | |  | | 0.008 |  | | -0.074 |  | | 85 |  | | -0.798 |  | | .427 |  |  |
| PE-Valence ✻ SOC vs. [PHY, CON] | | | 0.012  [0.002; 0.022] | |  | | 0.005 |  | | 0.114 |  | | 85 |  | | 2.303 |  | | .024 |  |  |
| PE-Valence ✻ PHY vs. CON | | | -0.003 [-0.012; 0.006] | |  | | 0.004 |  | | -0.036 |  | | 85 |  | | -0.724 |  | | .471 |  |  |
|  | *Note.* Linear mixed-effects model fit by maximum likelihood; dependent variable: learning rates derived from the valence model; learning rates for positive and negative prediction errors (PE, within subject factor PE-Valence); Stress group (SOC = social-evaluative stress [*n* = 29], PHY= physical stress [*n* = 30], CON = control [*n* = 29]) split in the contrasts SOC vs. [PHY,CON] and PHY vs. CON; *B* = unstandardized beta coefficient; *CI* = 95 % confidence interval;  *SE* = standard error of *B*; *b* = standardized beta coefficient; *df* = degrees of freedom. | | | | | | | | | | | | | | | | | | |  | |

**Group comparison of learning rates**

**Associations of valence bias score with stress response.**

As was to be expected, both measured components of the stress response, i.e. change in negative affect (∆AFF, post-stress T2_AFF_ - baseline T1_AFF_) and the cortisol response (∆CORT, post-stress T2_CORT_ - baseline T1_CORT_) share common variance (*ρ*_∆AFF,∆CORT_ = .31, *p*= .003). In order to test the effect of one component on the valence bias score (BIAS, (α_PE+_ - α_PE−_)/(α_PE+_ + α_PE−_)) independently of the other, a combined rank regression BIAS ~ ∆AFF + ∆CORT + TIME was calculated additionally. Neither the change in negative affect nor the change in cortisol could predict the valence bias score independently of the other stress component (*b*_ΔAFF_ = 0.054, *t*_85_ = 0.960, *p* = .340, *b*_ΔCORT_ = 0.016, *t*_85_ = 1.683, *p* = .096).

Within the subsamples of the three stress groups neither change in negative affect nor cortisol change could predict the valence bias score (all beta weights of the rank regression BIAS ~ ∆AFF as well as BIAS ~ ∆CORT + TIME are not significant, Table S6).

| *Table S6. Rank regression for the valence bias score predicted by the change in negative affect and cortisol change* | | | | | | | | | | | | | | | | | | |
| --- | --- | --- | --- | --- | --- | --- | --- | --- | --- | --- | --- | --- | --- | --- | --- | --- | --- | --- |
|  | | **Social Stress** | | | | |  | **Physical Stress** | | | | |  | **Control** | | | | |
|  | | **∆AFF** | |  | **∆CORT\| TIME** | |  | **∆AFF** | |  | **∆CORT\| TIME** | |  | **∆AFF** | |  | **∆CORT\| TIME** | |
| **Valence Bias Score** | *b* | .021 |  |  | .003 |  |  | .393 |  |  | .030 |  |  | -.199 |  |  | .045 |  |
|  | *p* | .749 |  |  | .789 |  |  | .089 |  |  | .200 |  |  | .275 |  |  | .067 |  |
|  | *n* | 29 |  |  | 29 |  |  | 30 |  |  | 30 |  |  | 29 |  |  | 29 |  |
| *Note*. Rank regression BIAS ~ ∆AFF and BIAS ~ ∆CORT + TIME. Valence bias score = (α_PE+_ - α_PE−_)/(α_PE+_ + α_PE−_), ∆AFF = change in negative affect (post-stress T2_AFF_ - baseline T1_AFF_), ∆CORT = Cortisol change (post-stress T2_CORT_ - baseline T1_CORT_), TIME = time of the day (morning vs. noon vs. afternoon); *b* = beta-weight of rank regression. | | | | | | | | | | | | | | | | | | |

| *Table S7. Rank regression of the affective recovery predicted by the valence bias score controlled for initial change in affect and modulated by the social stress group* | | | | | | | | | | |  | |
| --- | --- | --- | --- | --- | --- | --- | --- | --- | --- | --- | --- | --- |
|  |  | *b* | | | *SE* | | *t* | | *p* | |  | |
| Intercept | | 0.140 |  | 0.055 | |  | 2.561 |  | .012 |  | |  |
| BIAS | | 0.238 |  | 0.124 | |  | 1.922 |  | .058 |  | |  |
| SOC vs. [PHY, CON] | | 0.124 |  | 0.081 | |  | 1.534 |  | .129 |  | |  |
| PHY vs. CON | | -0.010 |  | 0.059 | |  | -1.171 |  | .865 |  | |  |
| ∆AFF | | 0.816 |  | 0.069 | |  | 11.831 |  | < .001 |  | |  |
| BIAS ✻ SOC vs. [PHY, CON] | | 0.322 |  | 0.189 | |  | 1.702 |  | .093 |  | |  |
| BIAS ✻ PHY vs. CON | | 0.005 |  | 0.139 | |  | 0.033 |  | .973 |  | |  |
| *Note*. Rank regression for the whole sample (*n* = 88, Affective Recovery ~ BIAS + ∆AFF + SOC vs. [PHY, CON] + PHY vs. CON + BIAS✻ SOC vs. [PHY, CON] + BIAS✻ PHY vs. CON). Affective recovery = post-stress T2_AFF_ - post-learning T3_AFF_; valence bias score/ BIAS = (α_PE+_ - α_PE−_)/(α_PE+_ + α_PE−_); ∆AFF = change in negative affect (post-stress T2_AFF_ - baseline T1_AFF_); *b* = beta-weight of rank regression. | | | | | | | | | |  | |  |

**Associations of valence bias score with affective recovery.**

**Associations of valence bias score with cortisol recovery.**

| *Table S8. Rank regression of the cortisol recovery predicted by the valence bias score controlled for initial change in cortisol* | | | | | | | | | | | | | | | | | |
| --- | --- | --- | --- | --- | --- | --- | --- | --- | --- | --- | --- | --- | --- | --- | --- | --- | --- |
|  | **Cortisol recovery \| ∆CORT** | | | | | | | | | | | | |  |  | | |
|  | **Social Stress** | | | |  | **Physical Stress** | | | |  | **Control** | | |  | **Whole sample** | | |
| **Valence Bias Score** | *b* | *p* | | *n* |  | *b* | | *p* | *n* |  | *b* | *p* | *n* |  | *b* | *p* | *n* |
|  | -0.58 | .706 | 29 | |  | 0.61 | | .663 | 30 |  | -1.31 | .252 | 29 |  | 0.19 | .804 | 88 |
|  |  |  | |  | |  |  |  |  |  |  |  |  |  |  | | |
| *Note*. Rank regression Cortisol Recovery ~ BIAS + ∆CORT. Cortisol recovery = post-stress T2_CORT_ - post-learning T3_CORT_; Valence bias score = (α_PE+_ - α_PE−_)/(α_PE+_ + α_PE−_); ∆CORT = stress-induced cortisol change (post-stress T2_CORT_ - baseline T1_CORT_); *b* = beta-weight of rank regression. | | | | | | | | | | | | | | | | | |

| *Table S9 a. Sample characteristics* | | | | | | | | | | | | | | | | | | | | |
| --- | --- | --- | --- | --- | --- | --- | --- | --- | --- | --- | --- | --- | --- | --- | --- | --- | --- | --- | --- | --- |
|  | **Social Stress** | | |  | | **Physical Stress** | | | |  | | **Control** | | | |  | | **Test** | | |
|  | *M* | *Md* | *SD* | |  | | *M* | *Md* | *SD* | |  | | *M* | *Md* | *SD* | |  | | *H(2)* | *p* |
| **Age** | 22.9 | 23 | 2.76 | |  | | 22.5 | 23 | 1.94 | |  | | 22.3 | 22 | 3.00 | |  | | 1.47 | .480 |
| **Self-esteem** | 6.44 | 6.75 | 1.02 | |  | | 6.30 | 6.42 | 0.94 | |  | | 6.02 | 6.25 | 0.93 | |  | | 5.03 | .080 |
| **SIAS** | 1.91 | 1.90 | 0.51 | |  | | 1.96 | 1.92 | 0.31 | |  | | 2.02 | 2.00 | 0.60 | |  | | 1.23 | .540 |
| **Cortisol baseline** | 8.04 | 7.07 | 5.22 | |  | | 6.17 | 4.88 | 4.17 | |  | | 7.30 | 5.09 | 5.89 | |  | | 1.74 | .419 |
| **Affective state baseline** | 1.27 | 1.25 | 0.33 | |  | | 1.28 | 1.25 | 0.39 | |  | | 1.40 | 1.25 | 0.39 | |  | | 3.21 | .201 |
| *Note.*  Sample characteristics for the three stress groups. *M* = mean; *Md* = median; *SD* = standard deviation; self-esteem assessed via averaged scores of the Self-Description Questionnaire (SDQ-III); SIAS = averaged score on the Social Interaction Anxiety Scale; *H* = Kruskal-Wallis Chi-squared. | | | | | | | | | | | | | | | | | | | | |

**Supplementary Tables**

| *Table S9 b. Sample characteristics* | | | | | | | | | | | | | | | | | | | |
| --- | --- | --- | --- | --- | --- | --- | --- | --- | --- | --- | --- | --- | --- | --- | --- | --- | --- | --- | --- |
|  |  | **Social Stress** | | |  |  | **Physical Stress** | |  |  | **Control** | |  | |  | **Test** | | |  |
|  |  |  |  |  | | |  | |  |  |  |  | |  | *H* | | *p* | |  |
| **Gender** | **female** | 21 |  |  | | | 20 |  |  | | 20 |  | |  |  | |  | |  |
|  | **male** | 8 |  |  | | | 10 |  |  | | 10 |  | |  | 0.3 (*df*=2) | | .861 | |  |
|  |  |  |  |  | | |  |  |  | |  |  | |  |  | |  | |  |
| **Time of day** | **morning** | 10 |  |  | | | 10 |  |  | | 10 |  | |  |  | |  | |  |
|  | **noon** | 11 |  |  | | | 10 |  |  | | 8 |  | |  |  | |  | |  |
|  | **afternoon** | 8 |  |  | | | 10 |  |  | | 12 |  | |  | 1.27 (*df*=4) | | .867 | |  |
| *Note*. Frequency distribution for gender and time of day of the measurement for the three stress groups; *H*= Pearson's Chi-squared test statistic | | | | | | | | | | | | | | | | | |  |  |
|  |  |  | | |  |  |  | |  |  |  | |  | |  |  |  | |  |

**References**

1. Vehtari, A., Gelman, A. & Gabry, J. Practical Bayesian model evaluation using leave-one-out cross-validation and WAIC. *Stat. Comput.* **27**, 1413–1432 (2017).
